# Editorial for Special Issue: Research on Plant Cell Wall Biology

**DOI:** 10.3390/cells11091480

**Published:** 2022-04-28

**Authors:** Christophe Dunand, Elisabeth Jamet

**Affiliations:** Laboratoire de Recherche en Sciences Végétales, Université de Toulouse, CNRS, UPS, Toulouse INP, 31320 Auzeville-Tolosane, France

Plant cells are surrounded by extracellular matrixes. These structures, also called cell walls, are highly variable between species and organs and are modified during plant development and upon environmental stresses. Primary cell walls are mainly composed of polysaccharides (cellulose, hemicelluloses, and pectins), but they also contain a small number of peptides and cell wall proteins (CWPs), presenting a large diversity [1,2]. The latter form part of the cell wall structure through covalent and non-covalent scaffolds or interactions with polysaccharides or CWPs, and they are critical players in cell wall dynamic processes [3,4,5]. They are also capable of sensing cell wall structure changes during development or in response to environmental constraints and, accordingly, convert them to signals, triggering appropriate physiological responses [6]. Secondary cell walls may contain aromatic polymers which contribute to cell wall rigidification and cell death for particular tissues [7].

The perception of biotic and abiotic signals via plasma membrane receptor-like kinases is well documented. By contrast, the sensing of cell wall integrity in order to balance and restore the cell wall structure is still puzzling [8,9,10]. Another fascinating subject concerns cell wall dynamics and constraints during lateral organ formation [11]. Indeed, cell walls which are necessary to maintain cell structure and integrity in response to cell turgescence need to be locally loosened to allow lateral organ emergence. To summarize, the plant cell wall is a solid, plastic, intelligent exoskeleton capable of sensing and responding to many types of stimuli [12,13,14].

This Special Issue has collected seventeen articles related to plant cell wall biology in a broad meaning, including fourteen research articles and three reviews. We are thankful to all the authors for their contributions, as well as to the *Cells* supporting team.

Several topics have been tackled: the diversity of cell wall polysaccharides and their roles during development [15,16,17]; the particular role of the arabinogalactan proteins (AGPs) [18,19]; the regulation of cell wall components biosynthesis, either at the transcriptional level [20] or at the biosynthesis level [21]; the role of the cell wall in signaling [22,23]; the remodeling of the cell wall in response to abiotic or biotic stress [24,25,26,27,28,29,30]; and a glance at the evolution of myxospermy in *Brassicaceae* [31].

Apart from the classical model plants such as *Arabidopsis thaliana* (dicotyledonous plant) [21,23,24], *Brachypodium distachyon* (monocotyledonous plant) [22], and *Populus sp* (woody plant) [20], a great variety of plant species has been studied: *Equisetum sp.* which were assumed to belong to the oldest extant genus among vascular plants [15]; *Miscanthus* x *giganteus,* as a promising cold-tolerant C4 plant for biomass production [26]; *Hordeum vulgare* and *Lolium multiflorum*, as crop or fodder plants [16,28]; *Craterostigma plantagineum*, known as the resurrection plant, exhibiting a unique cell wall folding mechanism conferring the capacity to withstand drought [29]; *Glycine max*, a plant of the *Leguminosae* (*Fabaceae*) family of great agronomic interest, exhibiting root border cells, secreting a mucilage with specific characteristics [25]; *Dionaea muscipula* and *Utricularia nelumbifolia*, which are carnivorous plants [17,18]; and *Bellis perennis*, an *Asteraceae* commonly used to study embryogenesis [30]. This large collection of plant species illustrates the interest of enlarging the perspective to tackle particular issues related to cell walls which cannot be studied using only classical model plants.

As mentioned above, the composition of the cell wall is highly complex and variable, depending on the cell type and on the environmental conditions. It is correlated to the cell architecture and function in the plant. This variability is possibly indefinite because of the variety of cell wall components and the presence of a large set of cell wall proteins acting on these components to tailor them for a best fit to the particular physiological situations encountered by plants. As an example, the function of (1,3;1,4)-β-D-glucans was reviewed by Chang et al. [15] who also recalled that these polysaccharides initially thought to be only synthesized by grasses (*Poaceae*), were also present in other taxa such as *Equisetum*, algae, lichens, fungi and bacteria. Immunocytochemistry using antibodies specific for cell wall epitopes has proven to be a very powerful tool to describe this diversity [32]. It has been used to characterize the distribution of (i) pectins and hemicelluloses in reproductive tissues of *U. nelumbifolia* [17], (ii) pectins, hemicelluloses and hydroxyproline-rich glycoproteins (HRGPs) in *G. max* root border cells, (iii) AGPs in ovules and anthers of *B. perennis* [30], as well as in the digestive glands of *D. muscipula* [18], (iv) pectins and AGPs in *H. vulgare* roots [28], hemicelluloses in *L. multiflorum* cell suspension cultures [16], and (vi) callose and pectins in the *A. thaliana* mutants *rol1-1* [23], and *ugt80A2* and *ugt80B1* [21], respectively.

The plasticity of the cell wall allows the cells to grow and respond to external stimuli. This property has been studied in different situations. van de Meene et al. [16] have followed the sequential appearance of different types of polysaccharides during the regeneration of the cell wall of *L. multiflorum* cell suspension cultures, thus demonstrating the importance of hemicelluloses in this process. Plachno et al. [17] have shown that the distribution of homogalacturonans and hemicelluloses in the reproductive female organs of *U. nelumbifolia* was not modified during pollination. Leszczuk et al. [30] have shown that low temperature induced modification in the distribution of AGPs during the development of *B. perennis* ovules and anthers. Bilska-Kos et al. [26] have demonstrated that the composition of *Miscanthus* x *giganteus* cell walls was modified upon cold exposure at the level of plasmodesmata, as revealed by transmission electron microscopy, infrared spectroscopy (FTIR) and biomechanical tests. The use of cellulose synthase inhibitors such as isoxaben and dichlobenil in cell suspension cultures have allowed Guerriero et al. [29] to tackle the question of the plasticity of *C. plantagineum* cell walls to overcome severe drought stress. Changes were observed at the protein level and a correlation could be established between the observed changes in protein accumulation and auxin levels. Milewska-Hendel et al. [28] have exposed *H. vulgare* roots to gold-nanoparticules and highlighted modifications in the pectin and AGP composition of the cell wall. Upon the infection of *G. max* roots by the *Phytophthora parasitica* oomycete, Ropitaux et al. [25] have shown that the infection was blocked by the presence of the root extracellular trap (RET), mainly composed of pectins, hemicelluloses and cellulose. Plachno et al. [18] have highlighted the modification of the AGPs distribution in the digestive glands of *D. muscipula* during the secretory cycle. Finally, Tsyganova et al. [27] have provided an overview of the present knowledge regarding the cell wall rearrangements occurring at the root microbial interface during the establishment of the *Rhizobium* infection thread. 

The changes in cell wall composition are regulated at different levels, from the transcriptional level to post-translational levels. In particular, Seyfferth et al. [20] have unraveled the role of the *Populus* PtERF85 transcription factor in the balance between xylem cell expansion and the formation of the secondary wall. Wu et al. [22] have shown the importance of wall-associated kinases (WAKs) for the response of *B. distachyon* to sodium salicylate or salt treatments: BdWAKs exhibited similar binding properties to acidic pectins as *A. thaliana* WAKs. Schumacher et al. [23] have shown that the defects observed in the *rol1-2* mutant impaired in the *Rhamnose synthase 1* gene was dependent on the cyclin-dependent kinase CDK8. The role of steroyl glycosyl transferases in the accumulation of rhamnogalacturonans I (RGI) and galactoglucomannans in the seed coat epidermal cells was demonstrated by Berger et al. [21]. In their review, Lamport et al. [19] have further developed their model of regulation of calcium exchange between the extracellular space and the cytosol: the so-called molecular pinball machine located at the plasma membrane requires an auxin-activated proton pump, AGPs, calcium channels and auxin-efflux PIN proteins. This mechanism could be involved in responses to mechanical stresses or oxidative stress mediated by reactive oxygen species (ROS). Thanks to an integrative study, Duruflé et al. [24] have studied a set of natural *A. thaliana* populations which exhibited an adaptative response to mild cold conditions. They have shown that the stems and leaves transcriptomes, as well as the cell wall proteomes and compositions were modified, thus demonstrating cell wall modifications. 

The last contribution questions the evolution of myxospermy in *Brassicaceae*, i.e., the ability of seeds to produce or extrude mucilage upon imbibition. Twenty-seven *Brassicaceae* species have been analyzed in detail and compared by Viudes et al. [31]. A high phenotypic diversity has been revealed although all the genes of the so-called *A. thaliana* mucilage secretory cell toolbox were found in most species. Once more, the *A. thaliana* model will not allow answering all the questions related to myxospermy.

The success of this Special Issue demonstrates the dynamism of the plant cell wall community. The high variability of the cell wall structures, compositions and roles is well illustrated, thus confirming the necessity to talk about “cell walls” rather than about “the cell wall”. It also appears that the diversity of the models studied is critical. Indeed, the usual ones only allow comprehending part of the issues, as exemplified by the *Brassicaceae* myxospermy. 
